# Correlation of Dual Colour Single Particle Trajectories for Improved Detection and Analysis of Interactions in Living Cells

**DOI:** 10.3390/ijms140816485

**Published:** 2013-08-08

**Authors:** Hendrik Deschout, Thomas Martens, Dries Vercauteren, Katrien Remaut, Jo Demeester, Stefaan C. De Smedt, Kristiaan Neyts, Kevin Braeckmans

**Affiliations:** 1 Laboratory of General Biochemistry and Physical Pharmacy, Ghent University, Harelbekestraat 72, B-9000 Gent, Belgium; E-Mails: hendrik.deschout@ugent.be (H.D.); thomas.martens@ugent.be (T.M.); dries.vercauteren@ugent.be (D.V.); katrien.remaut@ugent.be (K.R.); jo.demeester@ugent.be (J.D.); stefaan.desmedt@ugent.be (S.C.D.S.); 2 Center for Nano- and Biophotonics, Ghent University, B-9000 Gent, Belgium; 3 Liquid Crystals and Photonics Group, Ghent University, Sint-Pietersnieuwstraat 41, B-9000 Gent, Belgium; E-Mail: kristiaan.neyts@ugent.be

**Keywords:** interaction, fluorescence microscopy, single particle tracking, colocalization, correlation, endosomal escape, diffusion

## Abstract

Interactions between objects inside living cells are often investigated by looking for colocalization between fluorescence microscopy images that are recorded in separate colours corresponding to the fluorescent label of each object. The fundamental limitation of this approach in the case of dynamic objects is that coincidental colocalization cannot be distinguished from true interaction. Instead, correlation between motion trajectories obtained by dual colour single particle tracking provides a much stronger indication of interaction. However, frequently occurring phenomena in living cells, such as immobile phases or transient interactions, can limit the correlation to small parts of the trajectories. The method presented here, developed for the detection of interaction, is based on the correlation inside a window that is scanned along the trajectories, covering different subsets of the positions. This scanning window method was validated by simulations and, as an experimental proof of concept, it was applied to the investigation of the intracellular trafficking of polymeric gene complexes by endosomes in living retinal pigment epithelium cells, which is of interest to ocular gene therapy.

## 1. Introduction

In the field of gene therapy, a lot of effort goes into the development of nanomedicines, with a size in the order of 100 nm, for the delivery of therapeutic nucleic acids to target cells [1]. The way such nanomedicines are processed inside these cells is one of the main determinants of their effectiveness. In order to optimize the performance of nanomedicines, it is therefore important to understand how they interact with the intracellular constituents, such as endosomes, that are involved in their transport and final fate. Fluorescence microscopy is the ideal tool to make this type of information available, by simultaneously recording multi-colour live-cell images of fluorescently labelled nanomedicines and intracellular organelles [2–5].

The most common way of investigating interactions in multi-colour images is by comparing pixel values between colours, for which different quantification methods exist [6–11]. However, these pixel based methods are very susceptible to false positives, *i.e.*, all labelled compounds closer together than the microscope resolution (usually 250 nm or more) will contribute to the overall colocalization in the image. Fluorescence Resonance Energy Transfer (FRET) offers an alternative that is not restricted by the resolution, but has a limited working range of 1–10 nm [12]. Another approach is looking for the colocalization of discrete objects, rather than individual pixel values [13–18]. The basic condition here is that the objects of interest can be identified as separate entities in the microscopy images. One possibility to quantify object based colocalization is to compare their intensity weighted centre positions to each other [19]. The objects are classified as colocalized when their intensity weighted centre positions are closer together than a user defined maximum distance. Another possibility to quantify object based colocalization is to calculate the spatial overlap of the objects in both images [16]. Just like FRET, these object based methods are better in excluding false positives than pixel based colocalization, since the object positions can be determined with a precision much better than the microscope’s resolution [20,21].

In live-cell imaging, or any other application that involves dynamic events, the objects of interest, such as proteins or organelles, might be mobile. Two objects that are moving past each other by coincidence could, therefore, be identified as being colocalized by either the pixel or object based methods. This can be especially problematic in case of very dense object populations. One potential solution is to perform dual colour Image Cross-Correlation Spectroscopy (ICCS) [22,23]. Two interacting objects that move together will give rise to correlated fluorescence intensity fluctuations between the two simultaneously recorded detection channels. Unfortunately, this method only provides information that is spatially averaged over the part of the image that is included in the analysis. A different solution that retains the spatial information is to look at trajectories of moving objects in dual colour time-lapse movies, as is done in Single Particle Tracking (SPT). When the intensity weighted centre positions in the trajectories of two objects are closer together than a user defined maximum distance for a significant amount of time (*i.e.*, in multiple consecutive images), this is a strong indication that they are truly interacting [24–26]. Alternatively, the recently proposed Trajectory Image Correlation (TrIC) method identifies interaction between objects by calculating the local image correlation around the trajectory positions, and therefore, has the advantage that it does not require a user defined maximum distance [27]. However, the TrIC method assumes that the objects are reasonably close together (*i.e.*, within a 21 by 21 pixel analysis area), and since the analysis is performed at each time point separately, objects passing each other by coincidence might still be identified as interacting. Recently, our group proposed another approach for investigating the interaction between moving objects based on the spatial correlation of their trajectories [28]. When the correlation between the trajectories exceeds a certain threshold value, the corresponding objects are considered to be interacting. Interestingly, as correlation is translation independent, it does not require a user defined maximum distance and offers the possibility to detect interactions at any distance within the image. This was shown to give more reliable results than in case of classic object based colocalization analysis.

However, an objective measure for the correlation threshold has not been determined. Also, as the published correlation method is based on calculating the correlation between complete trajectories, it performs suboptimal in case trajectories are not completely correlated. For instance, intracellular motion can exhibit variable mobility, including immobile phases that inherently do not correlate. Another example is photobleaching of fluorescent labels, which degrades the localization precision in the trajectories, in turn affecting their correlation. There is also the possibility of transient interactions that take place during only a short time span, restricting the correlation to only a part of the trajectories. If the uncorrelated part of the trajectories in these situations is sufficiently large, the correlation determined from all positions in the trajectories will not exceed the correlation threshold, despite (transient) interaction being present. A method that can identify correlation in smaller segments of the trajectories with an objectively determined correlation threshold is, therefore, required. Here, such a method is presented, based on a scanning window approach in which the correlation is calculated over a limited number of positions within the trajectories. The optimal size of the window and the correlation threshold value are selected according to criteria that account for the localization precision in the trajectories and the mobility of the objects. The scanning window method is verified by simulations and applied to investigate the intracellular trafficking of polymeric gene complexes inside endosomes of living cells.

## 2. Theory

### 2.1. Identifying Interaction by Correlated Motion

As mentioned in the Introduction, we have recently proposed a new approach to identify interaction [28]. Instead of looking for colocalization in terms of a maximum distance, interaction between two objects is assumed to result in trajectories whose positions are correlated in time. Consider two sequences of images in different colours acquired at time points *t_i_* (with *i* = 1, …, *l*). The observed motion trajectory *A* of an object in one colour is given by (*x_A_*(*t_i_*), *y_A_*(*t_i_*)), and the observed motion trajectory *B* of an object in the other colour is given by (*x_B_*(*t_i_*), *y_B_*(*t_i_*)). The Pearson correlation coefficient ρ (≤1) between the *x*-coordinates of both trajectories can be calculated from:

(1) $$\rho =\frac{\sum_{i=1}^{l}\left(x_{A}(t_{i})-\langle x_{A}\rangle )(x_{B}(t_{i})-\langle x_{B}\rangle \right)}{\sqrt{\sum_{i=1}^{l}{\left(x_{A}(t_{i})-\langle x_{A}\rangle \right)}^{2}\sum_{i=1}^{l}{\left(x_{B}(t_{i})-\langle x_{B}\rangle \right)}^{2}}},$$

with <*x_A_*> and <*x_B_*> the average *x*-coordinates of the trajectories *A* and *B*, respectively. The same definition applies to the *y*-coordinates. From now on, we will only consider the *x*-coordinates as the theory equally applies to the other dimensions. Define σ*_A_* and σ*_B_* as the localization precisions with which *x_A_*(*t_i_*) and *x_B_*(*t_i_*), respectively, were determined. Besides various experimental noise sources, the localization precision is essentially determined by the number of detected photons and their spatial distribution in the image [20,21]. Define σ_o_ as the overlay precision with which both colour images are aligned, which can be calculated as the standard deviation of the differences between identical positions in the images after overlay [26].

**Figure 1:**
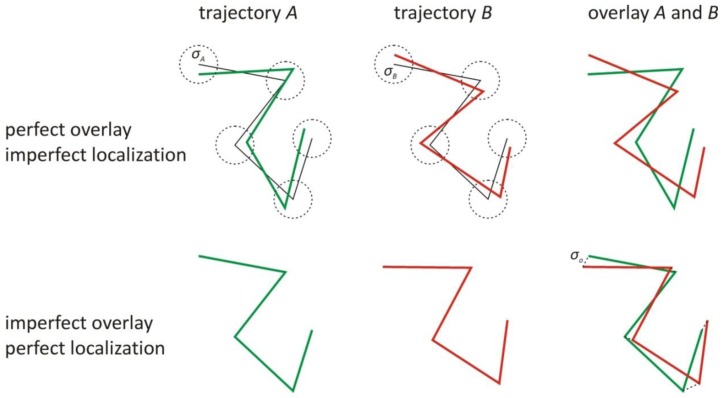
The effect of the localization and overlay precision on the observed trajectories of interacting objects. The localization precision σ*_A_* and σ*_B_* of the positions in the observed trajectories *A* (green) and *B* (red), respectively, are defined as the standard deviation (dotted circles) of the observed positions around the positions of the true trajectories (black). The overlay precision σ_o_ between the images is defined as the standard deviation of the differences (dotted lines) between identical positions in the images after overlay.

The effect of σ*_A_*, σ*_B_* and σ_o_ on the correlation ρ between the trajectories is illustrated in Figure 1, showing that, even if both objects are interacting, perfect correlation will not be obtained. This means that the computed correlation coefficient ρ < 1 should have a *p*-value smaller than 0.05, to make sure that it reflects true correlation rather than being obtained by coincidence under the null hypothesis that there is actually no correlation. However, a condition based on the *p*-value alone would mean that there is 5% chance of getting false positives in case of non-correlated trajectories. To reduce this probability, a correlation threshold ρ_min_, defined as the minimum correlation that is expected in case of correlated trajectories, can be imposed. As will be explained below, the ρ_min_ threshold value depends on σ*_A_*, σ*_B_* and σ_o_, as well as on other trajectory properties.

### 2.2. Correlation Threshold

For a certain localization and overlay precision, the correlation threshold ρ_min_ is defined as the minimum value of the correlation coefficient of interacting objects with a *p*-value smaller than 0.05. Although the localization precision may vary to some extent along a trajectory, we will assume that it remains constant, as motivated in Section 2.3. First, consider the situation of an equal localization precision σ = σ*_A_* = σ*_B_* in both trajectories and a perfect overlay precision σ_o_ = 0. For Brownian or linear motion, which is common in live-cell imaging, it can be shown that the expected correlation ρ between trajectories with *l* positions is completely determined by the relative localization error *r* (see Appendix):

(2) $$r=\frac{\sigma }{S},$$

where *S* is the mean step length in the trajectories, which can be estimated as:

(3) $$S=\frac{1}{2l}\sum_{i=2}^{l}\left(|x_{A}(t_{i})-x_{A}(t_{i-1})|+|x_{B}(t_{i})-x_{B}(t_{i-1})|\right).$$

The expected value of the observed correlation ρ is thus identical for all trajectory pairs with *l* positions and relative localization error *r*, which means that the same correlation threshold ρ_min_ can be used for all these trajectories.

It can be shown that the same applies to the general and more realistic case of σ*_A_* ≠ σ*_B_* and σ_o_ ≠ 0 (see Appendix). In this case, however, the localization precision σ in Equation (2) should be calculated according to:

(4) $$\begin{array}{l} \sigma^{2}\\ =-\frac{\text{var}(x_{A})+\text{var}(x_{B})-{\sigma_{A}}^{2}-{\sigma_{B}}^{2}-{\sigma_{\text{o}}}^{2}}{2}\\ +\frac{\sqrt{{\left(\text{var}(x_{A})+\text{var}(x_{B})\right)}^{2}+{\left({\sigma_{A}}^{2}-{\sigma_{B}}^{2}\right)}^{2}-2\left(\text{var}(x_{A})-\text{var}(x_{B})\right)({\sigma_{A}}^{2}-{\sigma_{B}}^{2})}}{2} \end{array}$$

where var(*x_A_*) and var(*x_B_*) are the variances of the *x*-coordinates in the trajectories *A* and *B*, respectively.

### 2.3. Scanning Window Concept

**Figure 2:**
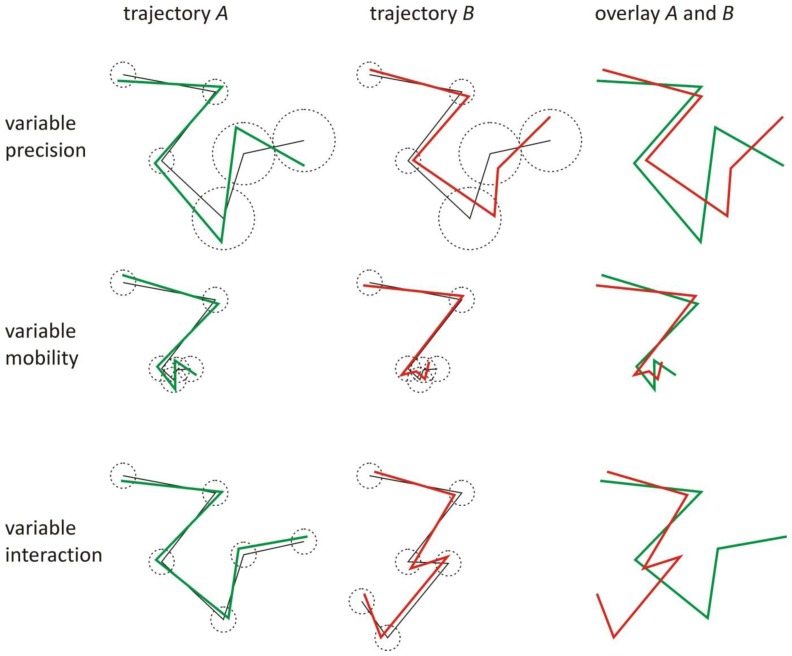
The effect of a time dependent mobility, a time dependent localization precision, or a time dependent interaction on the observed trajectories of interacting objects. The localization precision of the positions in the observed trajectory *A* (green) and *B* (red) is defined as the standard deviation (dotted circles) of the observed positions around the positions of the true trajectories (black). When one part of the trajectories exhibits low localization precision, the local relative localization error is large, degrading the correlation in that part. Also, when one part of the trajectories exhibits low mobility, the local relative localization error is large, which in turn degrades the correlation in that part. When the objects do not interact in one part of the trajectories, there is no correlation in that part.

In many circumstances, such as live-cell imaging, objects usually exhibit a variable mobility. When a certain part of the trajectories exhibits low mobility, the local mean step length *S* is smaller than the value over the entire trajectory. From Equation (2), it immediately follows that the local relative localization error *r* increases, which in turn decreases the correlation in this part of the trajectories. The same effect can be caused by a locally lower localization precision (*i.e.*, locally higher values of σ*_A_* and σ*_B_*), as can be seen from Equations (2) and (4). Another effect that can cause a change in correlation along the trajectories is the presence of transient interactions, such as binding and unbinding events. These different situations are illustrated in Figure 2. Thus, it is clear that assessing interaction by evaluating the correlation over the entire trajectories may not be optimal.

**Figure 3:**
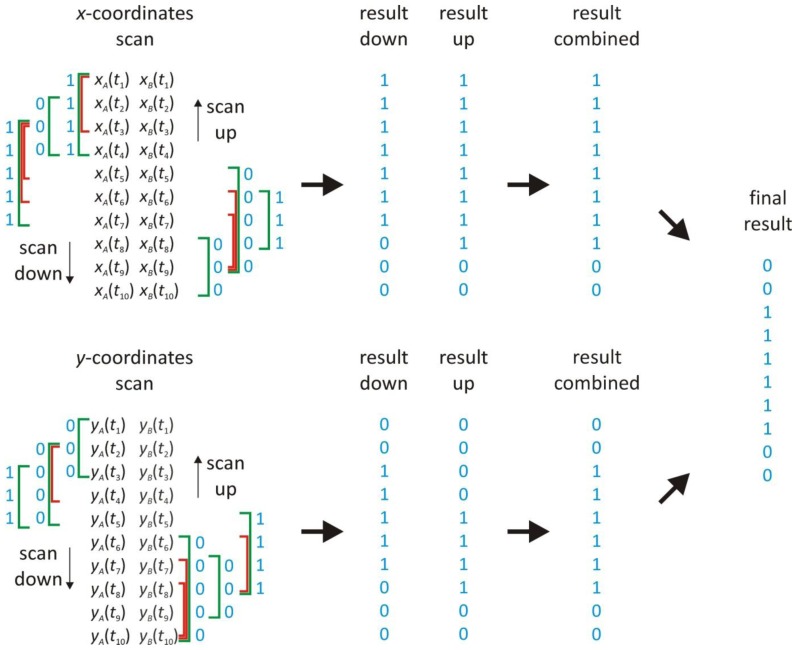
An illustration of the scanning window method. A trajectory *A* and a trajectory *B* are scanned by windows in two directions (up and down) for both the *x*- and *y*-coordinates independently. For each position, the window size starts at *w* = 3, and the probability *P* is calculated in the window (see Table 1). If *P* < 0.8 (red window), an extra position is included in the window, until an optimal window size with *P* ≥ 0.8 is found (green window) for which the correlation is calculated. If the correlation has a *p*-value smaller than 0.05 and is larger than the threshold ρ_min_ of the window (see Table 2), the positions in the window are assumed to interact (symbolized by binary values 1). If this is not the case, the positions are considered not to interact (symbolized by binary values 0). The results of the different windows and of both scans are combined according to the logical OR operation. The results of both coordinates are finally combined according to the logical AND operation.

One obvious solution to this problem lies in identifying correlation in smaller parts of the trajectories to which the framework of Section 2.2 can be applied. This idea leads to the scanning window method, as illustrated in Figure 3. Basically, the correlation is calculated in small overlapping subsets of trajectory positions, *i.e.*, in a window that is scanned along the trajectories. If the observed correlation in a window has a *p*-value smaller than 0.05 and is larger than the threshold ρ_min_ for that window, the objects are considered to be interacting in that window. The threshold ρ_min_ depends on the size of the window and the local relative localization error *r* (cfr. Section 2.2).

This raises the important question of what is the optimal window size. On the one hand, the window should be as small as possible in order to have the best temporal resolution and to ensure that the variation in relative localization error is minimal. On the other hand, the window should include a sufficient number of positions in order to detect correlation with sufficient statistical significance. Consider correlated trajectories and define *P* as the probability to observe a correlation with a *p*-value smaller than 0.05 inside a window with length *w*. Similar to the correlation threshold ρ_min_, this probability *P* depends on the relative localization error *r*. The optimal window length is then defined as the smallest *w* for which *P* becomes larger than a user defined value. Since the window size cannot be smaller than 3, each position will be evaluated in at least 3 different windows (except at the trajectory extremities). The probability that the correlation in at least one of those windows has a *p*-value smaller than 0.05 is given by 1 − (1 − *P*)^3^. A probability of more than 0.99 is achieved by *P* = 0.8, which is the threshold value for *P* used throughout this study.

### 2.4. Numerical Determination of ρ_min_ and P

The values of the correlation thresholds ρ_min_ (see Section 2.2) and the values of the probabilities *P* to identify the optimal window length (see Section 2.3) were obtained by simulating correlated trajectory pairs that represent windows of different sizes with different relative localization errors. The simulations were performed in the Matlab programming environment (The Mathworks, Natick, MA, USA). First, one-dimensional trajectories were simulated for each combination of trajectory length *w* and relative localization error *r* from a set of pre-defined values (*i.e.*, *w* = 3, 4, ..., 200 and *r* = 0.01, 0.02, ..., 1.00). The number of simulated trajectories *N_w_* depended on the trajectory length *w*, so that the total amount of positions from all trajectories together was approximately 10^6^ in all cases (e.g., for *w* = 10, the number of trajectories was 10^5^). The type of motion was chosen to be Brownian motion, since it is common on a microscopic scale, and because unrelated Brownian trajectories on average do not exhibit correlation. The diffusion coefficient was taken to be *D* = 1 μm^2^/s and the time interval between subsequent positions was τ = 0.1 s, resulting in a one-dimensional mean step of $S=\sqrt{2D\tau }=0.447\;\mu \text{m}$. The normally distributed step of the Brownian trajectories was simulated by the Matlab function *randn*. From each simulated trajectory, two correlated trajectories were extracted by separately adding two normally distributed values to each position of the simulated trajectory, again using the Matlab function *randn*. The standard deviation of the normal distribution for the extra value is the localization precision σ *= rS* (cfr. Equation (2)).

Subsequently, the correlation ρ between both trajectories is calculated, using the Matlab function *corrcoef* together with its corresponding *p*-value. For each value of *w* and *r*, let *N_w,p_* be the number of trajectory pairs that are correlated with a *p*-value smaller than 0.05. Then, *P* = *N_w,p_*/*N_w_* is the probability of finding a statistically significant correlation ρ in case of interacting objects. The results are partially shown in Table 1. The minimum value of the statistically significant correlations is selected as the threshold correlation ρ_min_ for a given *w* and *r*, as partially shown in Table 2. Note that for the smallest trajectory lengths *w* sometimes a correlation ρ smaller than zero was found (anti-correlation) with a *p*-value larger than 0.05. These correlations were treated as if they were not statistically significant. Also, note that the values of ρ_min_ do not always increase with the trajectory length *w*. When the trajectories are too short, only high enough correlations are statistically significant. Only from the point where the trajectories are long enough so that all correlations become significant (*i.e.*, *P* = 1) does ρ_min_ increase with *w*. The practical use of Tables 1 and 2 is explained in Section 2.5.

### 2.5. Scanning Window Method

The main input for the scanning window method consists of the trajectory *A* given by (*x_A_*(*t_i_*), *y_A_*(*t_i_*)) and the trajectory *B* given by (*x_B_*(*t_i_*), *y_B_*(*t_i_*)) at the time points *t_i_* (with *i* = 1, 2, ..., *l*). Another required input is the localization precision σ*_A_* and σ*_B_* of trajectory *A* and *B*, respectively, calculated within the window as it is scanned along the trajectories, and the overlay precision σ_o_ between the images (see Section 5.4 for information on how these values can be determined experimentally).

Consider first the *x*-coordinates of the trajectories *A* and *B*. The scan starts at *x_A_*(*t_i_*) and *x_B_*(*t_i_*), with a window of size *w* = 3, which thus covers the *x*-coordinates from *t*_1_ to *t*_3_. The relative localization error *r* in that window is calculated, according to Equations (2)–(4). For the relative localization error *r* and the window length *w* = 3, the probability *P* can be derived from Table 1, after rounding the value of *r* to the nearest tabulated value. For example, *r* = 0.045 is rounded to 0.05, and the corresponding row in Table 1 shows *P* = 0.653 (for *w* = 3). If the window has a probability *P* ≥ 0.8, it is considered to be the optimal window. If the window has a probability *P* < 0.8, it is extended to a size *w* = 4, covering the *x*-coordinates from *t*_1_ to *t*_4_. In the same manner, the probability *P* is calculated in the new window. This procedure is repeated until the optimal window size is reached for which *P* ≥ 0.8. In case the window size would become larger than both trajectories *A* and *B*, the calculation is aborted as correlation cannot be determined with sufficient certainty.

Having determined the optimal window size *w* and the local relative localization error *r*, the correlation threshold ρ_min_ can be determined from Table 2, again after rounding the value of *r* to the nearest tabulated value. Next, the correlation ρ between *x*-coordinates from both trajectories within the window is calculated according to Equation (1), together with the corresponding *p*-value. If the *p*-value is smaller than 0.05, and the correlation ρ is larger than the correlation threshold ρ_min_, all *x*-coordinates in the window are assigned a binary value 1 (see Figure 3). In all other cases, a binary value 0 is assigned to all *x*-coordinates in that window.

This procedure is repeated, starting at the next positions *x_A_*(*t*_2_) and *x_B_*(*t*_2_). The *x*-coordinates of the trajectories *A* and *B* are further scanned, position by position, until *x_A_*(*t_l−_*_2_) and *x_B_*(*t_l−_*_2_) have been reached. Except near the start and the end of the trajectory, the positions are evaluated at least three times by different windows. Therefore, for each position there are at least three binary values, indicating that correlation was found or not within a particular window. If correlation was found at least one time, the position is flagged as being correlated. This results in a list of binary values that identify the positions where the scan found correlation (see Figure 3). The same scanning procedure is repeated in the other direction, starting from *x_A_*(*t_l_*) and *x_B_*(*t_l_*) and moving towards the start of the trajectory. The results from both scanning directions are combined so that a position is correlated if it was flagged in one of both scanning directions (see Figure 3).

The identical scanning window procedure as described above is applied to the *y*-coordinates. Afterwards, interaction is assigned to a position if correlation was found in each dimension (see Figure 3). The result is a list of binary values that identify the positions where the objects were found to interact.

## 3. Results

### 3.1. Validation by Simulations

The performance of the scanning window method was verified with simulated pairs of two-dimensional Brownian motion trajectories, as explained in Section 5.1. Brownian motion was chosen, not only because it is common on a microscopic scale, but also because random Brownian motion trajectories are not expected to be correlated. A number of different situations were considered (see Table 3), for each of which 1000 trajectory pairs with length *l* = 20 and time interval τ = 0.1 s between successive positions were simulated.

**Figure 4:**
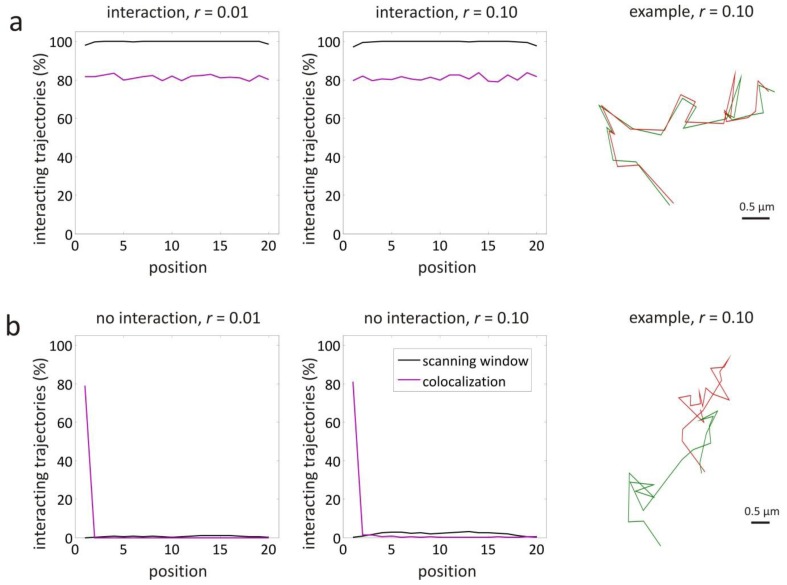
Validation simulations for interaction and no interaction. The percentage of 1000 pairs of simulated Brownian motion trajectories where the scanning window method has found interaction is shown for each position along the trajectories (black line), in case of (**a**) interaction, and (**b**) no interaction. All simulated trajectories have a length *l* = 20, a diffusion coefficient *D* = 1 μm^2^/s, and a time interval τ = 0.1 s between successive positions. The localization precision was chosen σ = 4.47 nm or σ = 44.7 nm, corresponding to a relative localization error of *r* = 0.01 or *r* = 0.10, respectively. The same trajectories were also analysed with an object based colocalization method with $d_{\text{max}}=1.65\sqrt{2}\sigma$ as maximum distance (purple line). On the right, example pairs of trajectories are shown for the case of *r* = 0.10.

The situation of complete interaction was investigated for a diffusion coefficient *D* = 1 μm^2^/s. The results are shown in Figure 4a, where for each position along the trajectories the percentage of trajectories where the scanning window method has detected interaction is shown. In case of high localization precision σ = 4.47 nm, corresponding to a relative localization error of *r* = 0.01 (cfr. Equation (2) with $S=\sqrt{2D\tau }=0.447\;\mu \text{m}$), the scanning window method correctly finds 100% of the time interaction at almost every position. Only at the trajectory start and end points, the method performs slightly worse, with interaction correctly detected 98% of the time. This can be explained by the smaller number of windows that correspond to the trajectory extremities (see Figure 3). For lower localization precision σ = 44.7 nm, corresponding to a relative localization error of *r* = 0.10, the scanning window method behaviour is essentially the same.

As shown in Figure 4a, these trajectories were also analysed with an earlier reported object based colocalization method that makes use of a maximum distance $d_{\text{max}}=1.65\sqrt{\sigma_{A}^{2}+\sigma_{B}^{2}+{\sigma_{\text{o}}}^{2}}$ to decide whether or not there is interaction at a particular position [26]. Here, $d_{\text{max}}=1.65\sqrt{2}\sigma$, considering σ*_A_* = σ*_B_* = σ and σ_o_ = 0. At almost all positions, the colocalization method finds interaction 81% of the time, for both relative localization errors *r* = 0.01 and *r* = 0.10.

Similarly, it was tested if the scanning window method can correctly detect the absence of interaction. This was investigated for a diffusion coefficient *D* = 1 μm^2^/s, the results of which are shown in Figure 4b. In case of high localization precision σ = 4.47 nm, corresponding to a relative localization error *r* = 0.01, the scanning window method finds less than 1% of the time interaction at almost all positions (*i.e.*, false positives). For lower localization precision σ = 44.7 nm, corresponding to a relative localization error *r* = 0.10, the method finds less than 3% false positives. The object based method with maximum distance $d_{\text{max}}=1.65\sqrt{2}\sigma$ finds that 81% of the trajectories are interacting at the first position, both in the case of *r* = 0.01 and *r* = 0.10, since the trajectories were simulated to start in the same position (see Section 5.1). From position 2, this percentage drops and remains below the percentage found with the scanning window method.

**Figure 5:**
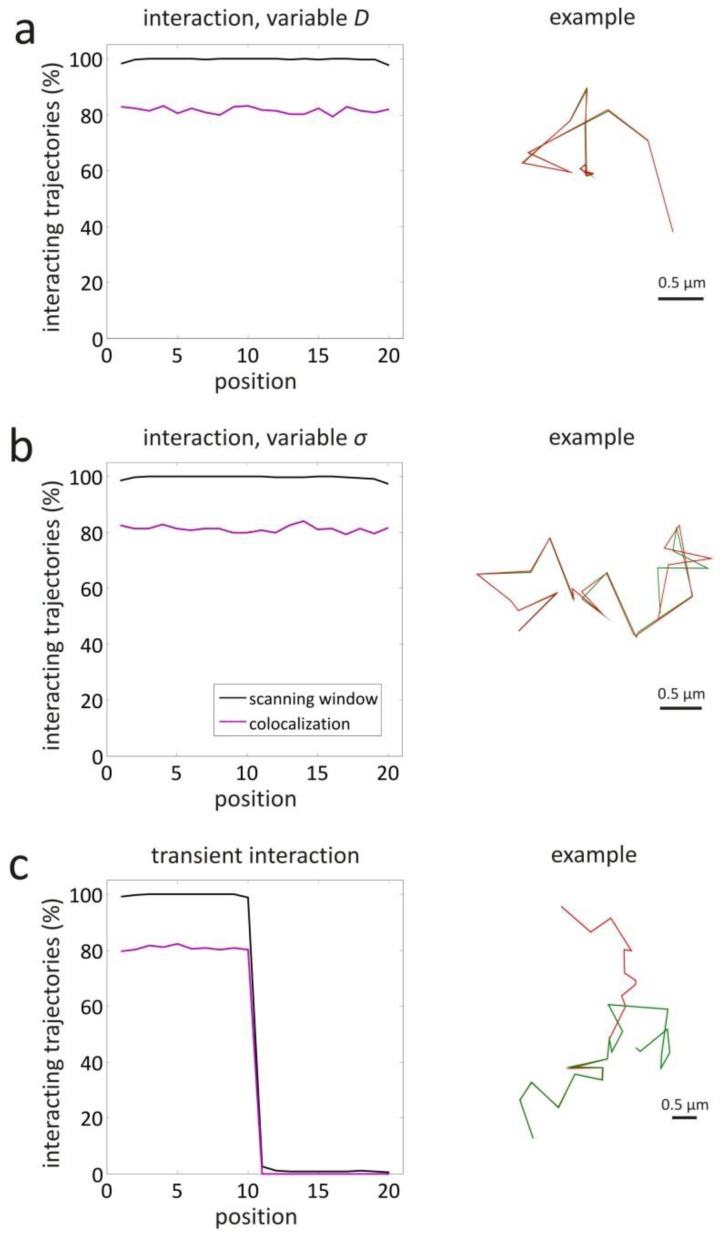
Validation simulations for variable diffusion coefficient, variable localization precision, and variable interaction. The percentage of 1000 pairs of simulated Brownian motion trajectories where the scanning window method has found interaction is shown for each position along the trajectories (black line), in case of (**a**) full interaction with localization precision σ = 4.47 nm, and a diffusion coefficient *D* = 1 μm^2^/s at positions 1–10 and *D* = 0.01 μm^2^/s at positions 11–20; (**b**) Full interaction with a diffusion coefficient *D* = 1 μm^2^/s, and a localization precision σ = 4.47 nm at positions 1–10 and σ = 44.7 nm at positions 11–20; (**c**) A diffusion coefficient *D* = 1 μm^2^/s, a localization precision σ = 4.47 nm, and interaction at positions 1–10 and no interaction at positions 11–20. All simulated trajectories had a length *l* = 20 and a time interval τ = 0.1 s between successive positions. The same trajectories were also analysed with an object based colocalization method with $d_{\text{max}}=1.65\sqrt{2}\sigma$ as maximum distance (purple line). On the right, example pairs of trajectories are shown.

Simulations were also carried out to evaluate the performance of the scanning window method in more complicated situations (representing the ones shown in Figure 2). The case of complete interaction with a variable diffusion coefficient was investigated for *D* = 1 μm^2^/s at positions 1–10 and *D* = 0.01 μm^2^/s at positions 11–20. This results in a corresponding local relative localization error *r* = 0.01 and *r* = 0.10, respectively, since the localization precision σ = 4.47 nm was constant at all positions. Thanks to the variable window size, the scanning window method finds interaction 100% of the time at most positions, as shown in Figure 5a. Only at the trajectory extremities, the method performs slightly worse, with interaction correctly detected 98% of the time. Although the object based colocalization method with maximum distance $d_{\text{max}}=1.65\sqrt{2}\sigma$ is not affected by differences in diffusion coefficient, only 81% of the trajectories is found to interact.

Complete interaction was also investigated with a variable localization precision σ = 4.47 nm at positions 1–10 and σ = 44.7 nm at positions 11–20. This results in a corresponding local relative localization error *r* = 0.01 and *r* = 0.10, respectively, since the diffusion coefficient *D* = 1 μm^2^/s was constant at all positions. The scanning window method finds 100% of the time interaction at most positions, as shown in Figure 5b. The object based colocalization method with maximum distance $d_{\text{max}}=1.65\sqrt{2}\sigma$ is not affected by differences in localization precision, so that 81% of the trajectories is found to interact at all positions.

Variable interaction was the last situation that was investigated, with the objects only interacting at positions 1–10 and not interacting at positions 11–20. The results are shown in Figure 5c, for a relative localization error of *r* = 0.01 (since *D* = 1 μm^2^/s and σ = 4.47 nm). Comparison to Figure 4 shows that the scanning window method performs as expected for the case of full interaction and no interaction. The transition from interaction to no interaction is almost perfectly detected at positions 9–11 with a resolution smaller than the expected window length *w* = 3 (see Table 1). Also, the object based colocalization method with maximum distance $d_{\text{max}}=1.65\sqrt{2}\sigma$ performs as expected, with interaction being found 81% of the time in the first half and virtually no interaction in the second half.

The simulations show that the scanning window method is capable of reliably identifying interaction, independent of the relative localization error. Even when parts of the trajectories are not correlated because of transient interactions, or exhibit low correlation because of a large local relative localization error, the scanning window method is still able to detect interaction when it takes place. An important benefit compared to the object based colocalization method is that the scanning window method is significantly less sensitive for false negatives that cannot be avoided by object based colocalization. Furthermore, it is much less sensitive to false positives in case of coincidental colocalization.

### 3.2. Intracellular Trafficking of Nanomedicines

In pharmaceutical research, nanomedicines such as polymeric gene complexes (polyplexes) are being developed for the delivery of therapeutic nucleic acids to target cells, such as retinal pigment epithelium (RPE) cells in the context of ocular gene therapy [29]. To improve therapeutic efficacy, it is of interest to have a detailed understanding of the postendocytic trafficking profile of polyplexes inside such cells [1]. In previous work, we have investigated the presence of nanomedicines in different types of endosomes in RPE cells as a function of time. This was done, using dual colour SPT on living RPE cells with one colour corresponding to the fluorescently labelled endosomes and the other to the fluorescently labelled polyplexes [28]. Trajectories of both polyplexes and endosomes were determined from the SPT images. The presence of polyplexes in endosomes was measured by determining the correlation, as defined in Equation (1), between the positions of the full trajectories in both colours. From here on, we will refer to this approach as the full trajectory method. This method was found to perform better than classic object based colocalization because it was less prone to find false negatives and insensitive to false positives due to coincidental colocalization. However, interactions might be overlooked when they only result in correlation over a limited part of the trajectories (see Section 2.3). This is especially relevant in the context of intracellular traffic, since such trajectories often exhibit immobile phases that do not correlate. Moreover, transient interactions such as the escape of a polyplex from an endosome or the transferral of the polyplex to another type of (unlabelled) endosome also give rise to trajectory pairs that are not completely correlated.

**Figure 6:**
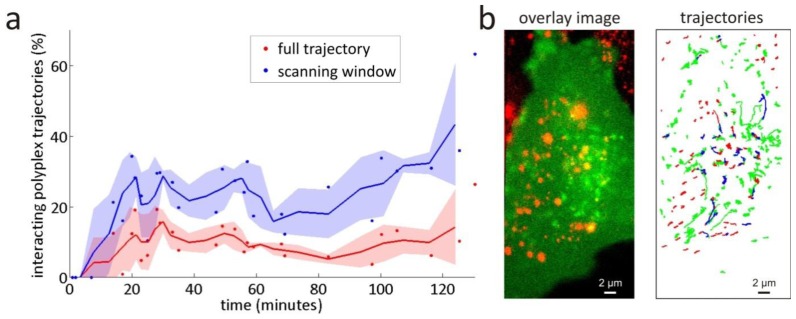
Interactions between endosomes and polyplexes measured by the scanning window method. (**a**) The percentages of polyplex trajectories that are interacting with a flotillin-2 type endosome trajectory inside living RPE cells at different time points after uptake of the polyplexes are shown. The dots represent the percentages for individual dual colour SPT experiments that each correspond to a different cell. The lines show the trend based on the running average of three subsequent experiments, and the shaded area above and below the lines represents the standard deviation of these averages. The red data corresponds to the full trajectory method and the blue data corresponds to the scanning window method. Using the scanning window method, a pair of trajectories was considered to interact if interaction was found in at least one window; (**b**) An overlay image and the corresponding trajectories of the dual colour SPT measurement at 53 minutes are shown (see Supplementary Movie 1). The endosomes have an eGFP label and are represented by green trajectories; the polyplexes have a Cy5 label and are represented by red trajectories. The windows where the scanning window method found interaction are indicated in blue. For this case, the window sizes *w* were situated between 3 and 16.

The scanning window method is, therefore, expected to perform better in the investigation of intracellular trafficking of nanomedicines than the full trajectory method, since it inherently is capable of detecting interaction in small segments of trajectories. First, as a negative control, dual colour SPT measurements of a mixture of non-interacting yellow-green and dark red fluorescently labelled 0.1 μm diameter beads undergoing free diffusion were analysed with the scanning window method to verify that no interactions are detected (see Appendix). Next, as a proof of concept, the scanning window method was applied to the dual colour SPT data of the postendocytic trafficking of polyplexes inside living cells (more details on the experiments can be found in Sections 5.2–5.4). The measured percentages of polyplex trajectories that are interacting in at least one window with a flotillin-2 endosome trajectory are shown in Figure 6a, together with the results obtained with the full trajectory method [28]. The data points show the percentages for individual dual colour SPT movies that each correspond to a different cell. Because of the variability between living cells, there is a strong variability in the corresponding percentages. The running average over three subsequent values is plotted to better indicate the observed trend. Comparison between the values from both methods shows that the scanning window method found at least two times more interaction than the full trajectory method. The same qualitative trend is found as for the scanning window method, indicating that the underestimation of the full trajectory method is systematic, and should thus always be accounted for.

Because of the variable localization precision and mobility in most trajectories in the live-cell dual colour SPT data, the relative localization error *r* is also variable along the trajectories, leading to different window sizes for the scanning window method (cfr. Section 2.5). For instance, the dual colour SPT movie recorded at 53 minutes (see Figure 6b) was analysed with window sizes *w* between 3 and 16. It should be noted that the localization precision in some of the endosome trajectories is particularly low (*i.e.*, values of the localization precision in the order of 200 nm). Combined with low endosome mobility, this can potentially lead to a large relative localization error *r* (*i.e.*, values of *r* in the order of 0.5). For such relative localization errors, the performance of the scanning window method is somewhat affected, leading to a higher probability to detect false positives (see Appendix).

**Figure 7:**
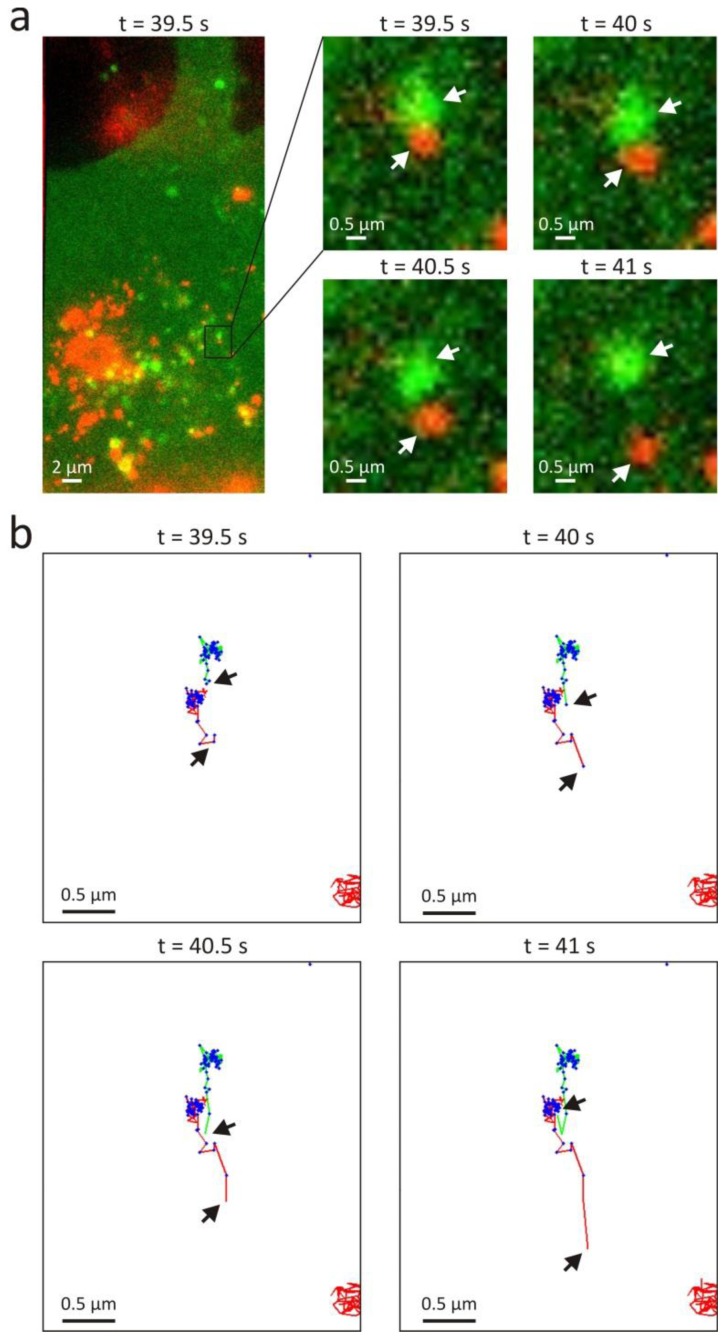
An example of transient interaction detected by the scanning window method. (**a**) On the left, an overlay image from a dual colour SPT experiment recorded 100 minutes after uptake of the polyplexes. The flotillin-2 type endosomes have an eGFP label (green) and the polyplexes a Cy5 label (red). On the right, a subregion shows a transient event where a polyplex and an endosome are at first exhibiting correlated motion, after which the polyplex moves away from the endosome (see Supplementary Movie 2); (**b**) The trajectories found in the subregion are coloured according to the fluorescent labels, and the interacting positions found by the scanning window method are indicated in blue. The scanning window method finds interaction until 40 s; afterwards it becomes apparent that both objects are not interacting anymore. For this case, the window sizes *w* were situated between 4 and 14.

Visual inspection of the trajectory pairs where the scanning window method only finds correlation in a part of the trajectories, suggests that this is mostly caused by either a low mobility or low localization precision in the other part of the trajectories (cfr. Figure 2). An exception is the clear example of transient interaction that was found in the dual colour SPT movie recorded at 100 min, as shown in Figure 7. Although this event could be interpreted as endosomal escape, it seems more likely that this is actually an endosomal fusion event where the polyplex is transferred to a different (unlabelled) type of endosome. The full trajectory method did not detect this transient interaction since the trajectories are not fully correlated. Interestingly, an earlier reported object based colocalization method [26] (see Section 3.1) also failed to identify this event because the endosome and the polyplex were separated further from each other than the user defined maximum distance.

## 4. Discussion

We have recently reported correlation between entire trajectories as a measure for the interaction between two dynamic species that is less prone to false positives and false negatives than classic object based colocalization [28]. However, this full trajectory method might not detect correlation in situations that are often present in live-cell imaging, such as changing mobility or transient interactions (see Section 2.3). Moreover, an objective measure for a threshold value of the correlation between trajectories of interacting objects was not determined. We, therefore, have developed a scanning window method, which allows spatial and temporal characterization of interaction by investigating the correlation in a window with a variable size that is scanned along the trajectories. The optimal window size depends on the local relative localization error *r* (cfr. Equation (2)) and is determined as the window size for which the probability *P* ≥ 0.8. The correlation threshold ρ_min_ for the optimal window depends in turn on both the window size and the local relative localization error *r*. The values of *P* and ρ_min_ can be determined from Tables 1 and 2, respectively.

The scanning window method was validated with simulated trajectory pairs (see Section 3.1). It was shown that the method is able to accurately identify interaction, independent of the relative localization error *r* (see Figure 4a). This should come as no surprise since Table 1 and 2 were determined from similar simulated trajectories of interacting objects (see Section 2.4). The scanning window method, however, was demonstrated to perform well in the case of no interaction as well (see Figure 4b). Only for a large relative localization error *r*, the percentage of false positives was found to increase slightly.

The performance of the scanning window method was also tested with simulated trajectory pairs that represent more complicated behaviour. In case of interaction along the entire trajectory, but with a changing diffusion coefficient, the scanning window method is still able to detect the interaction (see Figure 5a), because of the variable window size that accounts for the changing relative localization error. For the same reason, the method also performs well when the localization precision changes along the trajectory (see Figure 5b). Interestingly, the scanning window method is very well capable of detecting transient interactions along trajectories. The point at which the transition from binding to unbinding or vice versa occurs, was retrieved with a resolution that is smaller than the window size (see Figure 5c).

As a comparison, the same simulated data was also analysed with an earlier reported object based colocalization method that makes use of a maximum distance to decide whether or not there is interaction at a particular position [26]. As shown in Figure 4a, this method was found to be sensitive for false negatives, *i.e.*, interaction is significantly underestimated. It is also more sensitive to false positives in case of coincidental colocalization, which can happen when two independent objects pass by close to each other (see the first position in Figure 4b). The transition from binding to unbinding seems to be determined with a resolution that is similar to the scanning window method (see Figure 5c). Nonetheless, transient events detected by the object based colocalization method should be interpreted with care, considering for instance, the possibility of coincidental colocalization (see the first and second position in Figure 4b). Thus, it is clear that the scanning window method is overall a more reliable and robust method to detect true interaction.

As a proof of concept, the scanning window method was applied to the trajectories of polyplexes and endosomes inside living cells, obtained by dual colour SPT experiments (see Section 3.2). When interaction was found in at least one window, the polyplex was considered to be residing in, or at least interacting with, the endosome. Compared to the previously published full trajectory method [28], the scanning window approach was better capable of detecting this interaction. This is because it is for instance not uncommon for endosomes to exhibit mobility that changes over time [30,31]. In addition, a variable localization precision can occur, e.g. when the fluorescent labels photobleach. Both issues cause a variable local localization precision *r* and thus, a variable correlation along the trajectories. Correlation might also be degraded due to imperfect trajectory determination, for instance, because of the difficulty to unambiguously track the objects in crowded environments that are often present in living cells. In some cases, mistakes are unavoidable, leading to trajectories that contain incorrect positions. When there is interaction, the parts of the trajectories that correspond to the interacting objects still correlate, and hence, are found to interact by the scanning window method. A decrease in the overall correlation might also be caused by transient interactions, such as the escape of the polyplexes from endosomes, a process that is vital for the functioning of the polymeric gene complexes [1].

Comparison of the scanning window method with the full trajectory method shows that the latter method misses at least half of the interactions (see Figure 6a). Since it only searches for correlation on the full trajectory scale, the conventional correlation method does not notice many of the trajectory pairs that only partly correlate, due to the reasons discussed above. Interestingly, the scanning window method was capable of detecting transient interactions like the one shown in Figure 7.

The scanning window method could be tested on other types of motion besides diffusion, and Tables 1 and 2 for the determination of *P* and ρ_min_ could be adjusted if required. In the specific case that the objects are undergoing different types of motion, trajectory analysis could first be applied to determine the trajectory segments that correspond to these types of motion [32], which could then be analysed separately.

## 5. Materials and Methods

### 5.1. Validations Simulations

The scanning window method was validated by simulations in Matlab. Different sets of 1000 pairs of two-dimensional Brownian motion trajectories with length *l* = 20 and time interval τ = 0.1 s between successive positions were simulated. The Brownian motion step in each dimension was simulated with the Matlab function *randn*, assuming a standard deviation equal to the mean step $S=\sqrt{2D\tau }$. In most sets, the diffusion coefficient was taken *D* = 1 μm^2^/s, resulting in *S* = 0.447 μm. The two trajectories of each simulated pair start at the same position, and remain identical as long as there is interaction, depending on the set. A normally distributed value was added to each coordinate of each trajectory separately, again using the Matlab function *randn*. The standard deviation of this normal distribution is the localization precision σ, which is equal for both trajectories. The values of the localization precision were either chosen σ = 4.47 nm or σ = 44.7 nm, in order to obtain a relative localization error *r* = 0.01 or *r* = 0.10, respectively, according to Equation (2). The overlay was taken to be perfect, *i.e.*, σ_o_ = 0. In one set, the localization precision was different in the first and second half of the trajectories. In another set, the diffusion coefficient was different in the two trajectory halves, both leading to local relative localization errors in the windows that are variable. The different conditions of each set of simulated trajectories are listed in Table 3. The scanning window method is applied to each pair of simulated trajectories, as explained in Section 2.5.

### 5.2. Live-Cell Sample Preparation

The preparation of the sample for the live-cell dual colour SPT experiments is described in detail elsewhere [28]. Briefly, ARPE-19 cells (retinal pigment epithelial cell line, ATCC number CRL-2302) were cultured in DMEM:F12 supplemented with 10% FBS, 2 mm l-glutamine, and 2% P/S. All cells were grown at 37 °C in a humidified atmosphere containing 5% CO_2_. The pGL4.13 plasmid was labelled with Cy5 using the Label IT Nucleic Acid Labeling Kit (Mirus Bio Corporation, Madison, WI, USA) according to the manufacturer’s instructions at a 1:2 (*v:w*) ratio of Label IT Tracker Reagent and plasmid. Polymeric gene complexes were obtained by adding a poly(*N*,*N*′-cystaminebisacrylamide 4-aminobutanol) (p(CBA-ABOL)) solution of 0.6 mg/mL to a plasmid solution of 0.05 mg/mL in a final mass ratio of 48/1 in 25 mm HEPES buffer pH 7.2 and vortexing the mixture for 10 s. ARPE-19 cells were seeded at a concentration of 220.000 cells per well on sterile MatTek coverslips (1.5)-bottom dishes (MatTek Corporation, Ashland, MA, USA). The next day, cells were transfected with plasmids coding for the eGFP construct eGFP-Flot2 using Lipofectamine according to the manufacturer’s description. Fresh polymeric gene complexes were diluted 5× in OptiMEM when added to the cells expressing fluorescent protein constructs, corresponding to 4 μg of Cy5-labeled plasmid. Intense contact with the cells was assured through repetitive pipetting at room temperature, allowing electrostatic adhesion of the polyplexes to the plasma membrane. Next, the cells were washed and imaged in fresh OptiMEM to chase the cell-associated fraction of polymeric gene complexes.

### 5.3. Experimental Set-Up

The dual colour SPT experiments were carried out on a custom-built laser widefield epi-fluorescence microscope set-up that is described elsewhere in detail [33]. Briefly, the microscope was a Nikon TE2000-E (Nikon, Brussels, Belgium) with a Nikon Plan Apochromat NA = 1.4 oil immersion 100× objective lens. eGFP was excited with a 100 mW Calypso 491 nm diode pumped solid state laser (Cobolt, Solna, Sweden) and Cy5 was excited with a 30 mW IQ1C 636 nm diode laser (Power Technology, Little Rock, AR, USA). The fluorescence light coming from the sample was collected again by the objective lens and sent through the side port of the microscope towards a Cascade II:512 electron multiplication charge coupled device (EMCCD) camera (Roper Scientific, Tucson, AZ, USA). A pair of achromat lenses was placed in between the camera and microscope side port for an extra 2× magnification of the image on the CCD chip so that one pixel corresponded to a distance of 89 nm in the sample. A dichroic mirror placed between both achromat lenses reflected the fluorescent light with a wavelength below 630 nm and transmitted the wavelengths above 630 nm. Accompanying mirrors and notch filters (AHF Analysentechnik, Tuebingen, Germany) guided the reflected and transmitted part of the fluorescence each to one half of the CCD chip. High-speed movies were recorded using the NIS Elements (Nikon, Brussels, Belgium) imaging software. The EMCCD camera was synchronized with an acousto-optical tunable filter to only illuminate the sample during the actual camera exposure time so as to minimize phototoxicity and photobleaching. The living cells were placed on the microscope in a stage top incubation chamber (Tokai Hit, Shizuoka, Japan), set at 37 °C, 5% CO_2_, and 100% humidity.

### 5.4. SPT Experiments and Analysis

Movies of 60 seconds were recorded on different time points at a speed of 2 frames per second and with an image acquisition time of 30 ms. For each movie, a different cell was selected for imaging in order to minimize photobleaching and phototoxicity, and to obtain information on a large population of cells. Cells were chosen, based on a relatively low expression level of eGFP-constructs to minimize the possibility of a disturbed cell functioning.

After recording the movies, the images in the two different colours (*i.e.*, with fluorescence light above and below 630 nm) were aligned using an affine transformation. The transformation parameter values were determined from an image of immobilized beads (TetraSpeck, Molecular Probes, Gent, Belgium) that are fluorescent in both colours. Image processing was performed in Matlab on all images for identification of the individual object spots, as explained in detail elsewhere [33]. The object locations were determined using an intensity weighted centre algorithm, as it was recently shown that it is more robust than the fitting of a Gaussian function in case of moving objects [34]. Using a nearest neighbour algorithm, these positions were used to reconstruct the trajectories. Since the objects are moving stochastically, their position during image acquisition is unknown, making it impossible to determine the exact localization precision for an individual localization event. However, for the intensity weighted centre algorithm, it is possible to calculate the localization precision σ_c_ that is expected on average [34]:

(5) $${\sigma_{\text{c}}}^{2}=2\frac{s^{2}+a^{2}/12}{N}+2\frac{81\;\pi b^{2}{\left(s^{2}+a^{2}/12\right)}^{2}}{4a^{2}N^{2}},$$

where *a* is the pixel size, *b*^2^ describes the variance of the local photon background, *N* is the total number of photons in the observed spot, and *s* is the standard deviation of the Gaussian approximation of the average object spot for diffusion with a diffusion coefficient *D* during image acquisition time Δ*t* [34]:

(6) $$s^{2}={s_{0}}^{2}+\frac{D\Delta t}{3{z_{0}}^{2}}{s_{0}}^{2}+\frac{D\Delta t}{3},$$

where *s*_0_ = 150 nm is the standard deviation of the Gaussian approximation of the spot in case the object is stationary and located in the focal plane, and *z*_0_ is defined by:

(7) $$z_{0}=\frac{4\pi n}{\lambda }{s_{0}}^{2},$$

with λ ≈ 550 nm the wavelength of light and *n* ≈ 1.33 the refractive index of the sample. Note that in Equation (6), it is assumed that the objects remain around the focal plane while they are being tracked, which is a reasonable approximation in our live-cell SPT experiments. The conversion factor between pixel values and photon numbers needed for determining *b*^2^ and *N* in Equation (5) is calculated according to a standard procedure that is explained elsewhere [34,35]. The diffusion coefficient *D* in Equation (6) is determined from the mean square displacements in the trajectory (or in the window scanned along the trajectory), which is standard practice [34,36]. Besides the localization precision, the overlay precision was determined as σ_o_ = 3 nm for all movies by an experimental procedure as reported before [34].

The scanning window method is applied to each pair of trajectories, as explained in Section 2.5. To restrict the calculation time, trajectory pairs that cannot realistically correspond to interacting objects are not considered, *i.e.*, at least one pair of positions from both trajectories should be within a distance of 500 nm from each other, in both the *x*- and *y*-direction. When the method finds at least one window with correlation, the trajectories are assumed to originate from objects that, at least temporarily, interact with each other. When there are different candidate trajectories in one colour that are correlated with a certain trajectory in the other colour, the pair with the highest number of correlated positions is retained.

## 6. Conclusions

We have developed the scanning window method for measuring the interaction between moving objects in dual colour microscope time-lapse images. Employing a scanning window along two trajectories in which the correlation between the positions is calculated, not only spatial but also temporal information about the interaction becomes available. The scanning window method was validated with simulations and applied to the trajectories of endosomes and polymeric gene nanoparticles in living cells. Interaction was more reliably found with the scanning window method than by simple correlation analysis over the entire trajectory at once, which in turn was already proven to perform more reliably than the classic object-based approach. The additional temporal information thus allows a more sensitive estimation of the interactions between objects, and moreover, provides a means to detect transient interaction events.

## Figures and Tables

**Table 1:** The probability *P* to observe a statistically significant correlation in a window with length *w* in a pair of trajectories coming from interacting objects with relative localization error *r*. The values were obtained from simulated completely correlated trajectories, for different lengths *w* = 3, 4, …, 200 and different relative localization errors *r* = 0.01, 0.02, ..., 1.00. The full table can be found in the Table S1.

Simulated values of the probability *P*						

	*w* = 3	*w* = 4	*w* = 5	*w* = 6	…	*w* = 200
***r*= 0.01**	0.97177	0.99992	1	1		1
***r*= 0.02**	0.90433	0.99872	1	1		1
***r*= 0.03**	0.82096	0.99532	0.99980	1		1
***r*= 0.04**	0.73582	0.99020	0.99950	1		1
***r*= 0.05**	0.65341	0.98112	0.99815	0.99982		1
**⋮**						
***r*= 1.00**	0.04623	0.07992	0.12200	0.19284		1

**Table 2:** The correlation threshold ρ_min_ is the minimum statistically significant correlation in a window with length *w* and local relative localization error *r* in a pair of trajectories coming from interacting objects. The values are obtained from the correlation coefficient distribution of simulated completely correlated trajectories, for different lengths *w* = 3, 4, …, 200 and different relative localization errors *r* = 0.01, 0.02, ..., 1.00. The full table can be found in the Table S2.

Simulated values of the correlation threshold ρ_min_						

	*w* = 3	*w* = 4	*w* = 5	*w* = 6	…	*w* = 200
***r*= 0.01**	0.99693	0.95043	0.97095	0.99242		0.99998
***r*= 0.02**	0.99692	0.95013	0.88554	0.89819		0.9999
***r*= 0.03**	0.99692	0.95003	0.88114	0.91113		0.99972
***r*= 0.04**	0.99692	0.95004	0.88418	0.87069		0.99965
***r*= 0.05**	0.99692	0.95001	0.87854	0.81552		0.99925
**⋮**						
***r*= 1.00**	0.99693	0.95002	0.87836	0.81141		0.77525

**Table 3:** The conditions for each set of simulated trajectory pairs for the validation of the scanning window method. Each set consists of 1000 pairs of Brownian motion trajectories with trajectory length *l* = 20 and time interval τ = 0.1 s between successive positions. The presence or absence of interaction, the diffusion coefficient *D*, the mean step *S*, and the localization precision σ are listed in function of the trajectory positions.

Trajectory parameters used for the validation simulations					

Situation	Position	Interaction	*D* (μm^2^/s)	*S* (μm)	σ (nm)
interaction, *r* = 0.01	1–20	yes	1	0.447	4.47
interaction, *r* = 0.10	1–20	yes	1	0.447	44.7
no interaction, *r* = 0.01	1–20	no	1	0.447	4.47
no interaction, *r* = 0.10	1–20	no	1	0.447	44.7
interaction, variable *D*	1–10	yes	1	0.447	4.47
	11–20	yes	0.01	0.0447	4.47
interaction, variable σ	1–10	yes	1	0.447	4.47
	11–20	yes	1	0.447	44.7
variable interaction	1–10	yes	1	0.447	4.47
	11–20	no	1	0.447	4.47

**Figure A1:**
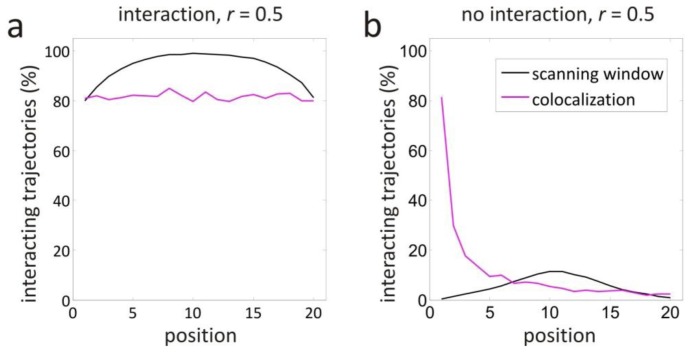
Validation simulations for interaction and no interaction in case of a large relative localization error. The percentage of 1000 pairs of simulated Brownian motion trajectories where the scanning window method has found interaction is shown for each position along the trajectories (black line), in case of (**a**) interaction, and (**b**) no interaction. All simulated trajectories have a length *l* = 20, a diffusion coefficient *D* = 1 μm^2^/s, and a time interval τ = 0.1 s between successive positions. The localization precision was chosen σ = 223.6 nm, corresponding to a relative localization error of *r* = 0.5. The same trajectories were also analysed with an object based colocalization method with $d_{\text{max}}=1.65\sqrt{2}\sigma$ as maximum distance (purple line).

**Figure A2:**
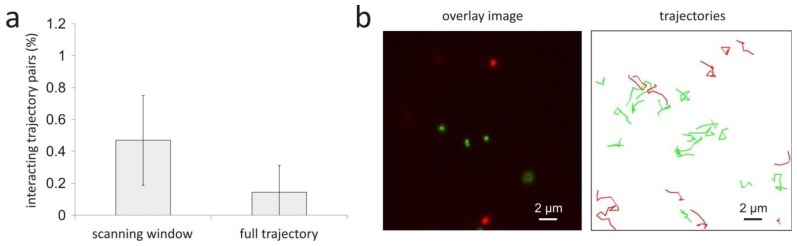
The scanning window method applied to dual colour SPT measurements on a mixture of yellow-green and dark red 0.1 μm diameter beads diffusing in water as an experimental negative control. (**a**) The average percentage of trajectory pairs in a dual colour SPT experiment that are found to interact by the scanning window and the full trajectory method. Both values were calculated from 10 dual colour SPT experiments and the error bars correspond to the standard deviation. All possible trajectory pairs were analysed, *i.e.*, there was no restriction on the distance between two trajectories. Using the scanning window method, a pair of trajectories was considered to interact when interaction was found in at least one window; (**b**) An overlay image and the corresponding trajectories of one dual colour SPT measurement are shown (see Supplementary Movie 3). The yellow-green beads are represented by green trajectories and the dark red beads are represented by red trajectories. The scanning window method did not find interactions between the trajectory pairs for this particular movie.

## Appendix

### 1. The Effect of the Localization and Overlay Precision on the Correlation

Consider a one-dimensional trajectory *X**_A_* and a one-dimensional trajectory *X**_B_*. The Pearson correlation *R* between both trajectories is given by:

(8) $$R=\frac{\text{cov}(X_{A},X_{B})}{\sqrt{\text{var}(X_{A})\text{var}(X_{B})}}.$$

The numerator is called the covariance and is defined as:

(9) $$\text{cov}(X_{A},X_{B})=\text{E}[(X_{A}-\text{E}[X_{A}])(X_{B}-\text{E}[X_{B}])],$$

where E[*X*] is the expected value of *X*. The denominator in Equation (8) is the square root of the product of two variances, defined by:

(10) $$\begin{array}{l} \text{var}(X_{A})=\text{E}[{\left(X_{A}-\text{E}[X_{A}]\right)}^{2}]\\ \text{var}(X_{B})=\text{E}[{\left(X_{B}-\text{E}[X_{B}]\right)}^{2}]. \end{array}$$

Assume now that the observed trajectories *x**_A_* and *x**_B_* deviate from the real trajectories *X**_A_* and *X**_B_*, respectively, because of experimental uncertainty:

(11) $$\begin{array}{l} x_{A}=X_{A}+\delta_{A}\\ x_{B}=X_{B}+\delta_{B}, \end{array}$$

with δ*_A_* and δ*_B_* deviations caused by the finite localization and overlay precision. The part coming from the localization precision follows a distribution around zero with standard deviation σ*_A_* and σ*_B_*, respectively. The deviations caused by the overlay process are not strictly defined, besides that their difference is following a distribution around zero with standard deviation σ_o_, which is called the overlay precision. For mathematical convenience, it is therefore assumed that δ*_A_* and δ*_B_* are distributed around zero with a standard deviation $\sigma_{A}^{\prime }=\sqrt{{\sigma_{A}}^{2}+{\sigma_{\text{o}}}^{2}/2}$ and $\sigma_{B}^{\prime }=\sqrt{{\sigma_{B}}^{2}+{\sigma_{\text{o}}}^{2}/2}$, respectively. Combining Equations (9) and (11), the covariance between *x**_A_* and *x**_B_* is given by:

(12) $$\text{cov}(x_{A},x_{B})=\text{cov}(X_{A},X_{B}).$$

The variance of *x**_A_* and *x**_B_* follow from Equations (10) and (11):

(13) $$\begin{array}{l} \text{var}(x_{A})=\text{var}(X_{A})+{{\sigma_{A}}^{\prime }}^{2}\\ \text{var}(x_{B})=\text{var}(X_{B})+{{\sigma_{B}}^{\prime }}^{2} \end{array}$$

The Pearson correlation ρ between the observed trajectories *x**_A_* and *x**_B_* is thus given by:

(14) $$\rho =\frac{\text{cov}(X_{A},X_{B})}{\sqrt{\text{var}(X_{A})\text{var}(X_{B})+{{\sigma_{B}}^{\prime }}^{2}\text{var}(X_{A})+{{\sigma_{A}}^{\prime }}^{2}\text{var}(X_{B})+{{\sigma_{B}}^{\prime }}^{2}{{\sigma_{A}}^{\prime }}^{2}}}.$$

Consider now the special situation σ*_A_**′* = σ*_B_**′* = σ, in this case the correlation becomes:

(15) $$\rho =\frac{\text{cov}(X_{A},X_{B})}{\sqrt{\text{var}(X_{A})\text{var}(X_{B})+\sigma^{2}\text{var}(X_{A})+\sigma^{2}\text{var}(X_{B})+\sigma^{4}}}.$$

Both correlations will be equal if the following condition for σ is fulfilled:

(16) $${{\sigma_{B}}^{\prime }}^{2}\text{var}(X_{A})+{{\sigma_{A}}^{\prime }}^{2}\text{var}(X_{B})+{{\sigma_{B}}^{\prime }}^{2}{{\sigma_{A}}^{\prime }}^{2}=\sigma^{2}\text{var}(X_{A})+\sigma^{2}\text{var}(X_{B})+\sigma^{4}.$$

This is a quadratic equation in σ^2^, with solution:

(17) $$\begin{array}{l} \sigma^{2}=-\frac{\text{var}(X_{A})+\text{var}(X_{B})}{2}\\ +\frac{\sqrt{{\left(\text{var}(X_{A})+\text{var}(X_{B})\right)}^{2}+4\left\{{{\sigma_{B}}^{\prime }}^{2}\text{var}(X_{A})+{{\sigma_{A}}^{\prime }}^{2}\text{var}(X_{B})+{{\sigma_{A}}^{\prime }}^{2}{{\sigma_{B}}^{\prime }}^{2}\right\}}}{2}. \end{array}$$

Using Equation (13) and considering the definitions of σ*_A_**′* and σ*_B_**′*, this can be rewritten as:

(18) $$\begin{array}{l} \sigma^{2}=-\frac{\text{var}(x_{A})+\text{var}(x_{B})-{\sigma_{A}}^{2}-{\sigma_{B}}^{2}-{\sigma_{\text{o}}}^{2}}{2}+\\ \frac{\sqrt{{\left(\text{var}(x_{A})+\text{var}(x_{B})\right)}^{2}+{\left({\sigma_{A}}^{2}-{\sigma_{B}}^{2}\right)}^{2}-2\left(\text{var}(x_{A})-\text{var}(x_{B})\right)({\sigma_{A}}^{2}-{\sigma_{B}}^{2})}}{2}. \end{array}$$

This expression is more useful than Equation (17), since the variances var(*X**_A_*) and var(*X**_B_*) cannot be determined experimentally.

In reality, the complete trajectories *x**_A_* and *x**_B_* are not known, only discrete positions *x**_A_*(*t**_i_*) and *x**_B_*(*t**_i_*) at different time points *t**_i_* (*i* = 1,2, …, *l*) are measured, from which the sample variances can be determined:

(19) $$\begin{array}{l} \text{var}(x_{A})=\frac{1}{l-1}\sum_{i=1}^{l}{\left(x_{A}(t_{i})-\langle x_{A}\rangle \right)}^{2}\\ \text{var}(x_{B})=\frac{1}{l-1}\sum_{i=1}^{l}{\left(x_{B}(t_{i})-\langle x_{B}\rangle \right)}^{2}, \end{array}$$

with <*x**_A_*> and <*x**_B_*> the average positions of the observed trajectories *x**_A_* and *x**_B_*, respectively:

(20) $$\begin{array}{l} \langle x_{A}\rangle =\frac{\sum_{i=1}^{l}x_{A}(t_{i})}{l}\\ \langle x_{B}\rangle =\frac{\sum_{i=1}^{l}x_{B}(t_{i})}{l}. \end{array}$$

### 2. Correlation between Trajectories of Interacting Objects

Consider a one-dimensional trajectory *X**_A_* of one object and a one-dimensional trajectory *X**_B_* of another object. Assume that both objects are interacting, resulting in identical trajectories, aside from a constant displacement *d*:

(21) $$\begin{array}{c} X_{A}=x\\ X_{B}=x+d. \end{array}$$

The observed trajectories *x**_A_* and *x**_B_* deviate from the real trajectories, because of experimental uncertainty:

(22) $$\begin{array}{c} x_{A}=x+\delta_{A}\\ x_{B}=x+d+\delta_{B}, \end{array}$$

with δ*_A_* and δ*_B_* deviations caused by the finite localization and overlay precision. As explained above, both can be assumed to be distributed around zero with equal standard deviation σ defined in Equation (17). According to Equation (15), the Pearson correlation between the observed trajectories *x**_A_* and *x**_B_* is thus given by:

(23) $$\rho =\frac{\text{cov}(x,x+d)}{\sqrt{\text{var}(x)\text{var}(x+d)+\sigma^{2}\text{var}(x)+\sigma^{2}\text{var}(x+d)+\sigma^{4}}}.$$

According to Equations (9) and (10), the covariance cov(*x*, *x* + *d*) and the variance var(*x*, *x* + *d*) are equal to:

(24) $$\begin{array}{c} \text{cov}(x,x+d)=\text{var}(x)\\ \text{var}(x+d)=\text{var}(x). \end{array}$$

This allows to rewrite Equation (23) as:

(25) $$\rho =\frac{1}{\sqrt{1+2\frac{\sigma^{2}}{\text{var}(x)}+{\left(\frac{\sigma^{2}}{\text{var}(x)}\right)}^{2}}}.$$

The correlation between observed trajectories of interacting objects is thus completely determined by the ratio of σ^2^/var(*x*). Assume for instance that the interacting objects are undergoing Brownian motion with diffusion coefficient *D*. If the trajectories are observed during a time *t*, the variance is given by [34]:

(26) $$\text{var}(x)=\frac{1}{3}Dt.$$

The mean step in the trajectory over a time interval τ < *t* is known to be [37]:

(27) $$S=\sqrt{2D\tau }.$$

Combining Equations (26) and (27) immediately results in:

(28) $$\text{var}(x)=\frac{t}{6\tau }S^{2}.$$

Another example is linear motion with velocity *v*. If the trajectories are observed during a time *t*, the variance is given by:

(29) $$\text{var}(x)=\frac{1}{12}v^{2}t^{2}.$$

The (mean) step in the trajectory over a time interval τ < *t* is:

(30) S=vτ.

Combining Equations (29) and (30) immediately results in:

(31) $$\text{var}(x)=\frac{t^{2}}{12\tau^{2}}S^{2}.$$

Linear and Brownian motion thus give rise to the following relationship between trajectory variance and mean step:

(32) $$\text{var}(x)=fS^{2},$$

where *f* is a factor that depends on the ratio *t*/τ between the observation time and time interval for the step. Inserting this expression in Equation (25) gives:

(33) $$\rho =\frac{1}{\sqrt{1+\frac{2}{f}{\left(\frac{\sigma }{S}\right)}^{2}+\frac{1}{f^{2}}{\left(\frac{\sigma }{S}\right)}^{4}}}.$$

In other words, for a certain ratio *t*/τ, the observed correlation between two interacting objects undergoing Brownian or linear motion is completely determined by the following ratio, termed the relative localization error:

(34) $$r=\frac{\sigma }{S}.$$

However, the mean step *S* cannot be determined experimentally. In reality, the actual trajectory *x* is not known, only discrete positions *x**_A_*(*t**_i_*) and *x**_B_*(*t**_i_*) different time points *t**_i_* (*i* = 1, 2, ..., *l*) are measured. In this case the time interval is given by τ = *t**_i_* − *t**_i_*_−1_ (*i* = 2, ..., *l*) and the total observation time by *t* = *l*τ, from which immediately follows that the ratio *t*/τ = *l*. From the trajectories the sample mean steps can be determined as:

(35) $$S_{AB}-\frac{1}{2l}\sum_{i=2}^{l}\left\{|x_{A}(t_{i})-x_{A}(t_{i-1})|+|x_{B}(t_{i})-x_{B}(t_{i-1})|\right\}.$$

These are estimations of the mean steps defined in Equations (27) and (30). All observed trajectories with length *l* of interacting objects that are undergoing Brownian or linear motion will thus have the same expectation value for the correlation if they have the same relative localization error *r*. This result is valid for all types of motion that fulfill the condition in Equation (32), *i.e.*, the variance of the trajectories of the interacting objects should be linearly related to the square of the mean step.

### 3. The Influence of a High Localization Error

In case of low localization precision or low mobility, the relative localization error *r* will be large (see Equation (34)). Simulations were performed to investigate the influence of a large relative localization error *r* = 0.5 on the performance of the scanning window method.

Two sets of 1000 pairs of two-dimensional Brownian motion trajectories with length *l* = 20 and time interval τ = 0.1 s between successive positions were simulated in the Matlab programming environment (The Mathworks, Natick, MA, USA). The Brownian motion step in each dimension was simulated with the Matlab function *randn*, assuming a standard deviation equal to the mean step $S=\sqrt{2D\tau }$. The diffusion coefficient was taken *D* = 1 μm^2^/s, resulting in *S* = 0.447 μm. In both sets, the two trajectories of each simulated pair start at the same position. The subsequent positions are also identical in the first set (*i.e.*, interaction), while they are independent from each other in the second set (*i.e.*, no interaction). A normally distributed value was added to each coordinate of each trajectory separately, again using the Matlab function *randn*. The standard deviation of this normal distribution is the localization precision *σ*, which is equal for both trajectories. The value of the localization precision was chosen σ = 223.6 nm, in order to obtain a relative localization error *r* = 0.5. The overlay was taken to be perfect, *i.e.*, σ_o_ = 0. The scanning window method is applied to each pair of simulated trajectories.

The results in the situation of complete interaction are shown in Figure A1a, where for each position along the trajectories the percentage of trajectories where the scanning window method has detected interaction is shown. The scanning window method finds 99% of the time interaction in the middle of the trajectories. Towards the trajectory extremities, the method performs worse, reaching 80% at the trajectory start and end point. This can be explained by the smaller number of windows that correspond to the trajectory extremities.

As shown in Figure A1a, these trajectories were also analysed with an earlier reported object based colocalization method that makes use of a maximum distance $d_{\text{max}}=1.65\sqrt{2}\sigma$ to decide whether or not there is interaction at a particular position [26]. At almost all positions, the colocalization method finds interaction 81% of the time.

Similarly, it was tested if the scanning window method can correctly detect the absence of interaction, the results of which are shown in Figure A1b. The scanning window method finds that less than 1% of the trajectories are interacting at the trajectory start and end points (*i.e.*, false positives). However, away from the trajectory extremities, the scanning window method performs worse, going up to 11% in the middle of the trajectories. The object based method with maximum distance $d_{\text{max}}=1.65\sqrt{2}\sigma$ finds that 81% of the trajectories are interacting at the first position, since the trajectories were simulated to start in the same position. From position 2, this percentage drops and becomes smaller than 10% from position 5.

For large relative localization errors, the performance of the scanning window method can thus be affected, leading to a somewhat higher probability to detect false positives. In this situation, the results of the scanning window method should thus be interpreted with care.

### 4. Negative Control Experiment

Dual colour SPT measurements were performed on a mixture of yellow-green and dark red fluorescently labelled 0.1 μm diameter beads (FluoSpheres, Molecular Probes, Gent, Belgium). Afterwards, the scanning window method was used to search for interaction between the bead trajectories. This provides a negative control, since no interaction is expected between the yellow-green and dark red beads.

The microscope sample was prepared by diluting the bead mixture in water and applying 5 mL between a microscope slide and a cover slip with a double-sided adhesive spacer of 120 μm thickness (Secure-Seal Spacer, Molecular Probes, Bleiswijk, The Netherlands) in between. The dual colour SPT experiments were carried out on a custom-built laser widefield epi-fluorescence microscope set-up that is described elsewhere in detail [33]. Ten movies of 6 seconds were recorded at a speed of 35 frames per second and with an image acquisition time of 6 ms. The camera frame rate was obtained by selecting a subregion on the CCD chip of 256 by 512 pixels. After recording the dual colour SPT movies, the images in the two different colours were aligned using an affine transformation, calculated from an image of immobilized beads (TetraSpeck, Molecular Probes, Gent, Belgium) that are fluorescent in both colours. The individual beads were identified in each image of all movies by image processing performed in Matlab, as explained in detail elsewhere [33]. After determining the bead positions by an intensity weighted centre algorithm, the bead trajectories were reconstructed by a nearest neighbour algorithm. The average localization precision for the intensity weighted centres was calculated as explained in detail elsewhere [34].

The scanning window method was applied to all possible pairs of trajectories. Note that no restriction was imposed on the distance between the trajectories, which is possible with the scanning window method because correlation is translation independent. Using the scanning window method, a pair of trajectories was considered to interact when interaction was found in at least one window. The average percentage of trajectory pairs in a dual colour SPT experiment that are found to interact by the scanning window is shown in Figure A2a, together with the results obtained with the full trajectory method [28]. The scanning window method identified only 0.5% of the trajectory pairs as interacting, and the full trajectory method performs even slightly better with only 0.1% of trajectory pairs detected as interacting. The scanning window method thus correctly predicts the absence of interaction.

## Supplementary Files

Supplementary File 1 Supplementary (ZIP, 5344 KB)
