# Supplementary material for: Correlation of Dual Colour Single Particle Trajectories for Improved Detection and Analysis of Interactions in Living Cells
Source: Int J Mol Sci. 2013 Aug 8;14(8):16485–514. doi: 10.3390/ijms140816485 (PMC3759922; doi:10.3390/ijms140816485)
Supplement: Supplementary File 1 — Supplementary (ZIP, 5344 KB) [file ijms-14-16485-s001.zip › ijms-35835-supplementary information.pdf]

Supplementary Information

Supplementary Movie 1. An overlay of two movies of a living RPE cell recorded at 53 min after uptake of the polyplexes for 60 s with a speed of 2 frames per second and with an image acquisition time of 30 ms. The green movie corresponds to the fluorescent label of the flotillin-2 type endosomes and the red movie corresponds to the fluorescent label of the polyplexes. Note that the contrast of the red movie was adjusted to include the dimmer objects.

Supplementary Movie 2. A subregion in the overlay of two movies of a living RPE cell recorded at 100 min after uptake of the polyplexes for 60 s with a speed of 2 frames per second and with an image acquisition time of 30 ms. The green movie corresponds to the fluorescent label of the flotillin-2 type endosomes and the red movie corresponds to the fluorescent label of the polyplexes. A transient event is shown where the polyplex and endosome are at first exhibiting correlated motion during the first 40 s, after which the polyplex moves away from the endosome.

Supplementary Movie 3. An overlay of two movies of a mixture of beads diffusing in water recorded for 6 s with a speed of 35 frames per second and with an image acquisition time of 6 ms. The green movie corresponds to the fluorescent label of the yellow-green 0.1 µm diameter beads and the red movie corresponds to the fluorescent label of the dark red 0.1 µm diameter beads.

Table S1. The probability P to observe a statistically significant correlation in a window with length w in a pair of trajectories coming from interacting objects with relative localization error r. The values were obtained from simulations of completely correlated trajectories for different lengths $w = 3, 4, \dots, 200$ and different relative localization errors $r = 0.01, 0.02, ..., 1.00$.

Table S2. The correlation threshold ρ_{min} is the minimum statistically significant correlation in a window with length w and local relative localization error r in a pair of trajectories coming from interacting objects. The values are obtained from the correlation coefficient distribution for simulated completely correlated trajectories for different lengths $w = 3, 4, \dots, 200$ and different relative localization errors $r = 0.01, 0.02, ..., 1.00$.

© 2013 by the authors; licensee MDPI, Basel, Switzerland. This article is an open access article distributed under the terms and conditions of the Creative Commons Attribution license (http://creativecommons.org/licenses/by/3.0/).
